# Early detection of anaemia in primary care with haemoglobinometry: ANHEMOG clinical trial protocol

**DOI:** 10.1186/s12875-021-01548-z

**Published:** 2021-10-08

**Authors:** Boris Trenado Luengo, Rosa García-Sierra, Maria Asunción Wilke Trinxant, Esther Díaz Mondelo, Ramon Miralles Baseda, Maria Magdalena Lladó Blanch, Maria del Pilar Montero Alia, Pere Toran-Monserrat

**Affiliations:** 1 Centre d’Atenció Primària Badalona Centre-Dalt la Vila. Gerència Territorial Metropolitana Nord, Institut Català de la Salut, Barcelona, Spain; 2 Multidisciplinary Research Group in Health and Society GREMSAS (2017 SGR 917), Barcelona, Spain; 3 Research Support Unit Metropolitana Nord, Primary care Research Institut Jordi Gol (IDIAPJGol), Barcelona, Spain; 4 Nursing Department, Faculty of Medicine. Universitat Autònoma de Barcelona, Barcelona, Spain; 5 Centre d’Atenció Primària Badalona Bufalà-Canyet. Gerència Territorial Metropolitana Nord, Institut Català de la Salut, Barcelona, Spain; 6 Departament de Geriatria, Hospital Universitari Germans Trias i Pujol, Barcelona, Spain; 7 Servei d’Atenció Primària Barcelonés Nord i Maresme, Gerència Territorial Metropolitana Nord, Institut Català de la Salut, Barcelona, Spain; 8 Centre d’Atenció Primària La Riera (Mataró 1), Institut Català de la Salut, Barcelona, Spain; 9 Department of Medicine, Faculty of Medicine, Universitat de Girona, Girona, Spain

**Keywords:** Anaemia, Red blood cell transfusion, Advanced practice nursing, Nurse case manager, Haemoglobinometry, Non-invasive haemoglobin measurement, Primary health care

## Abstract

**Abstract:**

**Background:**

Detecting, treating and monitoring anaemia has a functional, social and economic impact on patients’ quality of life and the health system, since inadequate monitoring can lead to more accident & emergency visits and hospitalizations. The aim of this study is to evaluate the impact in the patient clinical outcomes of using haemoglobinometry to early detect anaemia in patients with chronic anaemia in primary care.

**Methods:**

Randomized controlled trial Capillary haemoglobin will be measured using a haemoglobinometer on a monthly basis in the intervention group. In the control group, the protocol currently in force at the primary care centre will be followed and venous haemoglobin will be measured. Any cases of anaemia detected in either group will be referred to the transfusion circuit of the reference hospital.

**Discusion:**

The results will shed light on the impact of the intervention on the volume of hospitalizations and accident & emergency (A&E) visits due to anaemia, as well as patients’ quality of life. Chronic and repeated bouts of anaemia are detected late, thus leading to decompensation in chronic diseases and, in turn, more A&E visits and hospitalizations. The intervention should improve these outcomes since treatment could be performed without delay. Improving response times would decrease decompensation in chronic diseases, as well as A&E visits and hospitalizations, and improve quality of life. The primary care nurse case manager will perform the intervention, which should improve existing fragmentation between different care levels.

**Trial registration:**

NCT04757909. Registered 17 February 2021. Retrospectively registered.

## Background

The World Health Organization defines anaemia as a condition in which haemoglobin (Hb) concentration is lower than 13 g/dl in men and 12 g/dl in women [1, 2]. The global prevalence of anaemia is 5–10% in patients aged 65 to 70 [3] and 15–25% in patients over 80. Moreover, prevalence ranges from 48 to 60% in patients presenting frailty criteria, according to data from the National Geriatrics Research Consortium and the Beverly Healthcare Data Warehouse [4].

Currently, many patients with chronic anaemia require periodic transfusions. While blood transfusions are safe, they are not exempt from producing adverse effects, so patients must be monitored during the procedure by trained providers who know how to detect and respond to any reactions that may arise and prevent negative clinical outcomes [5] [6]. Several studies recommend making personalized indications for blood transfusions based on the patient’s clinical context, pathologies and preferences rather than exclusively on Hb and haematocrit levels in order to reduce the risks inherent to transfusions. In asymptomatic patients without cardiovascular risks, transfusion is rarely needed until Hb drops below 7–8 g/dl or haematocrit falls between 21 and 24%, although there are some clinical circumstances in which is not recommended to let Hb drop that low for an extended period, or the optimal transfusion strategy is unclear. For example, in patients with coronary artery disease, it has been found that a restrictive transfusion strategy (in most cases, with the limit at 9 mg/dl) is linked to a higher risk of experiencing another cardiac ischemic event, while a personalized, liberal transfusion strategy lowers that same risk [7–11], however, the observed results of a recent clinical trial suggests that there may be merit to a restrictive strategy [7].

Chronic anaemia can lead to multiple decompensations, not just as a result of the disease itself, but also due to its contribution to the adverse development of other chronic pathologies. The prevalence of anaemia in patients with heart failure (HF) is approximately 37% [8], although it can range from 9 to 69.6% depending on the patient’s characteristics. Patients with HF and anaemia present greater cardiovascular risk and higher mortality [2, 3, 8]. Iron deficiency in patients with low ejection fraction is associated with decreased long-term survival and lower quality of life [9].

As for patients with chronic obstructive pulmonary disease (COPD), the incidence of anaemia is estimated to be about 12%, although studies vary, reporting from 7.5–33% depending on patient selection criteria. Moreover, in this group of patients, there is also an association between low haematocrit levels and increased mortality, more hospitalizations and longer average duration of hospital stays [1] . Low Hb in patients with emphysema is also directly linked to mortality. Several studies on the intensive care received by patients with severe COPD and anaemia observed a statistically significant reduction in minute ventilation and the work of breathing, with discharge of respiratory muscles and higher mortality [1, 10]. A study by Sarkar et al. (2015) on COPD patients admitted to intensive care units found that extubation in patients with Hb levels above 12 g/dl was more suitable than in patients with Hb levels below 12 g/dl. In addition to the direct impact anaemia has on the evolution of respiratory diseases and mortality, it also affects quality of life due to the way it limits exercise capacity [1].

Other diseases, such as upper and lower gastrointestinal bleeding, can also lead to chronic anaemia and require transfusions. For those without curative care, therapy most often consists of transfusions to reduce mortality and rebleeding, although they must be based on clinical judgement [11, 12].

Detecting, treating and monitoring anaemia has a functional, social and economic impact on patients’ quality of life and the health system [4], since inadequate monitoring can lead to more A&E visits and hospitalizations [13]. In general, haematocrit and Hb levels are measured in at least 8 ml of venous blood extracted through venipuncture. The blood is transported to a lab where it is measured using automated equipment. There are also portable systems available known as haemoglobinmeters that provide haemogram results in five seconds using just a drop of capillary blood. Clinically speaking, these devices have proven to be accurate and precise in post-anaesthesia care units, intensive care units, hospital A&E and anaemia screening before blood donation [14–16].

Anaemia is a complex clinical issue that often requires coordination between different levels of care. A Nurse Case Manager, is a nurse with a training similar to an Advanced Practice Nurse, and play a crucial role in the communication between primary health care and the hospital level, to ensure the continuity of care and avoiding the defragmentation of the health system [17–19]. The use of haemoglobinmeters by nurse case managers in primary care, on patients with chronic diseases that require regular transfusions would facilitate early detection of low Hb levels and swift inclusion in the adequate hospital circuit for dispensing treatment. This, in turn, would decrease decompensations in chronic diseases requiring extended hospitalization and improve the volume of A&E visits due to this cause [17–19].

The purpose of the present paper is to describe a study protocol for a randomized controlled trial of an intervention directed to early detect anaemia in patients with chronic anaemia through the use of haemoglobinometry in primary care, and thus reduce the number of hospital admissions compared with the standard surveillance of anaemia.

## Methods/design

### Research question

Monthly capillary Hb measurement with a hemoglobinometer improves patient outcomes compared to standard venipuncture monitoring in chronically anemic patients?

### Main objective

Compare the number of hospitalizations due to anaemia of patients receiving monthly capillary Hb measurements and those receiving standard venipuncture monitoring.

### Secondary objectives

Compare the number of hospital A&E visits due to anaemia of patients receiving monthly capillary Hb measurements and those receiving standard monitoring.

Compare changes in quality of life of patients receiving monthly capillary Hb measurements and those receiving standard monitoring.

Assess satisfaction with monthly haemoglobinometer measurements.

### Hypothesis

The research hypothesis is that periodic measurements taken by the nurse in primary care using the haemoglobinometer will decrease the number of A&E visits and hospitalizations and improve quality of life as compared to standard venous Hb measurements.

### Design

Randomized controlled trial number NCT04757909.

### Sample

#### Setting

Catalan Health Institute primary care centres in Santa Coloma de Gramenet, Badalona and Mataró (Barcelona, Spain).

Inclusion criteria

1. Patients over 40 who had two or more transfusions of packed red blood cells due to chronic anaemia in a year.
2. Patients’ whose primary care health provider is the Catalan Institute of Health.

Exclusion criteria

1. Patients with kidney failure receiving haemodialysis treatment.
2. Patients receiving palliative care.
3. Patients presenting moderate to severe cognitive impairment, with a score of 5 or higher on the Pfeiffer questionnaire and who do not have a caretaker capable of detecting signs of decompensation in the patient.

**Fig. 1:**
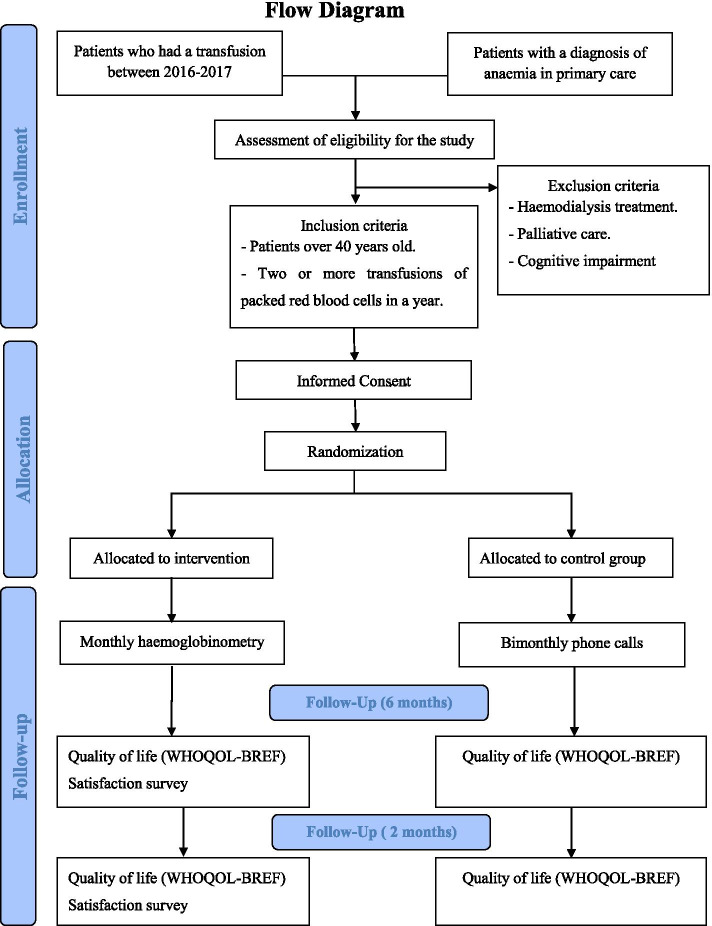
CONSORT Study Flow Chart

The study procedure is explained in the Consolidated Standards of Reporting Clinical Trial (CONSORT) flow chart (Fig. 1) [20].

#### Sample size

The sample size was calculated at 138 patients, 69 in both the control and intervention group, thus making it possible to detect a difference in hospitalization of 25% (60% in the control group and 35% in the intervention group, based on the disposable information in the database of the health provider). This sample size also takes into account an alpha risk of 0.05, a beta risk below 20% and a 10% dropout rate, with bilateral comparison. The arcsin approach was used for this calculation.

#### Assigning patients to interventions

Patients will be randomly assigned to the control or intervention group using a list of numbers with a random computer-generated allocation to control or intervention in an adjacent column.

#### Data collection, management and analysis

Data will be collected at the monthly visits of participants in the intervention group and the bimonthly visits of participants in the control group.

### Intervention

The Hb levels of participants in the intervention group will be taken once a month with a Veri Q® haemoglobinometer, that showed good performance data, in accuracy test compared with the reference value from Nihon Kohden MEK-6420 (R2 = 0.9881) and with the Sysmax reference value (R2 = 0.9968) [21]. If a patient’s haemoglobin level falls below their reference value, the nurse case manager will activate the transfusion circuit by scheduling an urgent referral to the day hospital, an outpatient service where short-term treatment and diagnostic tests are performed. The interdisciplinary team at this unit will prescribe and administer the transfusion and handle the hospital discharge, in a specific circuit between health levels created to this intervention.

The WHOQOL-BREF questionnaire on quality of life will be administered at the first visit and six and 12 months.

To know the satisfaction with the intervention, a single item survey will be administered upon inclusion in the study and six and 12 months. The question is “to what extent are you satisfied with your anemia control”, and the possible answers are not satisfied, satisfied and very satisfied.

An ad hoc satisfaction survey on the intervention will be administered upon inclusion in the study and six and 12 months.

Participants in the control group received standard monitoring, the frequency of which is variable, since it is determined by clinical criteria. The data will be collected by reviewing patients’ computerized clinical records and a bimonthly phone call to determine how many times they have been hospitalized and visited the A&E during the study period. The WHOQOL-BREF questionnaire on quality of life will be administered at the first visit and six and 12 months of inclusion in the study. Data will also be collected for the control group.

### Criteria for suspending or modifying the interventions assigned to each subject in the trial

Participants can leave the study at any time and may withdraw consent.

They will be excluded from the trial in the following cases:

1. Death.
2. They wish to leave the study.
3. Palliative care is initiated and transfusions are rejected.

During the trial, patients in the control group may not have their capillary Hb levels measured.

No risks are anticipated for patients by participation in this study.

### Statistical analysis

Statistical analyses will be conducted on a blinded intention-to-treat basis, with all participants who were initially randomised into the study included where data are available for each measurement time point.

Univariate descriptive statistics:

The main numerical variables will be described by measures of centrality and dispersion or absolute and relative frequencies in the case of categorical variables. Univariate comparisons between the intervention group and control group will be performed using mean comparison tests (T-test or similar) or distribution comparison tests (Chi-squared or similar).

Predictive model:

The result variables will be considered discrete quantitative variables. Multiple linear regression will be used to model the data collected, taking those that present significant differences in the previously calculated univariate models as independent variables, in addition to the demographic and clinical variables considered relevant.

### Validity and reliability/rigour

To ensure rigour in the collection of quantitative data, it will be included in a pseudonymized database so that participants cannot be identified, without any additional data, which will be recorded separately using techniques that guarantee it cannot be traced. Data will be stored in a local repository that only the researchers have access to. Participants will be randomly assigned to the groups to avoid any selection bias.

To improve compliance with intervention protocols, the clinical trial supervisor will prepare a report each semester detailing whether or not follow-up visits are being held and properly recorded in the data collection form.

Throughout the trial, if patients who are not in the transfusion circuit are identified, they will be included therein based on the consensus of hospital and primary care professionals.

### Expected results

#### The main result variables are

- Number of hospitalizations due to anaemia.
- Number of A&E visits due to anaemia.
- Time elapsed between Hb measurement and circuit activation.
- Time elapsed between Hb measurement and when the transfusion is performed.
- Time elapsed between Hb measurement and return home.

The specific measurement variable is the Hb level taken with the Veri-Q® haemoglobinometer once a month.

Other variables are:

- Satisfaction with monthly anaemia monitoring: taken at six and 12 months. Ad hoc questionnaire.
- Quality of life: measured at the start of the study and six and 12 months. WHOQOL-BREF questionnaire [22, 23].
- Prescribed medication that might alter Hb or haematocrit levels.
- Signs and symptoms of anaemia at monthly visits (dyspnoea, asthenia, palpitations, bleeding, and chest pain).

Socio-demographic and clinical data will be collected from both groups for comparison, including age, sex, educational level, type of anaemia, primary care centre, the physician responsible for monitoring anaemia, cognitive status, inclusion in the case management program, number of haemogram controls performed via venipuncture.

## Discussion

Primary care professionals experience difficulties in monitoring haemoglobin levels in patients with chronic anaemia due to delays in receiving haemogram results, sometimes of up to 12 h. Using a haemoglobinometer in patients with chronic diseases and chronic anaemia would allow healthcare providers to detect the need for transfusions or other treatment and activate the hospital circuit at the corresponding care level early and quickly, thus preventing decreased quality of life and worse prognosis in patients, reducing decompensations and optimizing health resources.

The haemoglobinometer is a portable, pocket-sized device that can be easily transported to patients’ home. It is very simple to use and can be operated by any health professional with minimal training [14–16] . The technique consists of pricking the patient’s finger, collecting a drop of capillary blood with a micropipette and depositing it on a test strip inserted into the device [24]. In short, it is a simple technique that requires minimal training and can be performed in a matter of minutes.

Moreover, in our setting there is no existing a specific and direct circuit to introduce patients who were detected anemia in primary care, the intervention test a procedure to avoid this difficulty and try to improve the defragmentation of the health system for these patients, nor have we seen evidence of such a circuit in other regions with similar a population.

Therefore, this study will allow us to implement and assess a transfusion circuit, the rapid response of hospital resources to perform transfusions, user satisfaction with this resource, any incidents that may arise and any improvements needed. The results of this study are expected to facilitate the implementation of an early-detection anaemia circuit and a quick inter-level response that improves care for patients with chronic anaemia. Disseminating the protocol will facilitate reproducibility in other regions.

The figure of the nurse case manager is essential to carrying out the treatment circuit since they are responsible for defragmenting the health system [17–19]. That is why they are in charge of initiating and monitoring the entire intervention process in this study and employing fluid communication with all the parties involved.

Early detection of anaemia contributes to improved quality of life in patients with the clinical profile outlines in this study since we expect to find greater satisfaction among those who are monitored with a haemoglobinometer than those who undergo venipuncture. This expectation is based on the easier and less invasive technique used to obtain the sample [16], as well as lower levels of emotional distress, anxiety and uncertainty that patients experience while waiting for results that will determine what interventions will be necessary since, with a haemoglobinometer, the results are available almost immediately upon taking the sample. Moreover, by monitoring anaemia with a haemoglobinometer, we predict a drop in A&E visits and hospitalizations, thus reducing patients’ discomfort and the risks associated with such visits, as well as the health expense they represent.

One of the main limitations of this study is recruitment difficulty. In recent years, there has been a tendency to limit transfusions, meaning it is possible that fewer patients than initially expected meet the inclusion criteria. If the recruitment objectives cannot be met, the recruitment region could be expanded to include other nearby geographical areas with similar socio-demographic and health characteristics.

If the results of the study are as expected, health institutions could evaluate the possibility of using a haemoglobinometer to monitor patients who frequently experience anaemia and adding a transfusion circuit to the reference day hospital. If the hypothesis is confirmed, the number of hospitalizations and A&E visits would be reduced and patients’ quality of life would improve. Future studies might also hypothesize that healthcare costs would decrease considerably as well. The regular use of a haemoglobinometer by nurse case managers in primary and home care would enable early detection and treatment of anaemia.

### Trial status

Protocol version number: 6.0, date: 1 September 2017. Date the recruitment began: 6 March 2018. The approximate date when recruitment will be completed: 31st December 2023.

## Data Availability

Only the Prinicipal Investigator and the clinical trial supervisor have access to the data. The results will be disseminated via peer reviewed publication and conference presentation. This protocol is written according to the SPIRIT guideline.

## Abbreviations

Hb: Haemoglobin
HF: Heart failure
COPD: Obstructive pulmonary disease
A&E: Accident & emergency

## References

[1] Sarkar M, Rajta PN, Khatana J (2015). Anemia in chronic obstructive pulmonary disease: prevalence, pathogenesis, and potential impact. Lung India.

[2] Sîrbu O, Floria M, Dascalita P, Stoica A, Adascalitei P, Sorodoc V, Sorodoc L (2018). Anemia in heart failure- from guidelines to controversies and challenges. Anatol J Cardiol.

[3] Wouters HJCM, van der Klauw MM, de Witte T, Stauder R, Swinkels DW, Wolffenbuttel BHR, Huls G (2019). Association of anemia with health-related quality of life and survival: A large population-based cohort study. Haematologica..

[4] Urrutia A, Sacanella E, Mascaro J, Formiga F (2010). Anemia en el anciano. Rev Esp Geriatr Gerontol.

[5] Bolton-Maggs PHB (2014). Bullet points from SHOT: key messages and recommendations from the annual SHOT report 2013. Transfus Med.

[6] SETS (2015). Guía sobre la transfusión de componentes sanguíneos y derivados plasmáticos. Sociedad Española de Transfusión sanguínea y Terapia Celular.

[7] Ducrocq G, Gonzalez-Juanatey JR, Puymirat E, Lemesle G, Cachanado M, Durand-Zaleski I (2021). Effect of a restrictive vs Liberal blood transfusion strategy on major cardiovascular events among patients with acute myocardial infarction and Anemia: the REALITY randomized clinical trial. JAMA.

[8] Comín-Colet J, Martín Lorenzo T, González-Domínguez A, Oliva J, Jiménez MS (2020). Impact of non-cardiovascular comorbidities on the quality of life of patients with chronic heart failure: A scoping review. Health Qual Life Outcomes.

[9] Wienbergen H, Pfister O, Hochadel M, Fach A, Backhaus T, Bruder O (2019). Long-term effects of iron deficiency in patients with heart failure with or without anemia: the RAID-HF follow-up study. Clin Res Cardiol.

[10] Gadre SK, Jhand AS, Abuqayyas S, Wang X, Guzman J, Duggal A (2020). Effect of Anemia on mortality in mechanically ventilated patients with chronic obstructive pulmonary disease. J Intensive Care Med.

[11] Odutayo A, Desborough MJR, Trivella M, Stanley AJ, Dorée C, Collins GS (2017). Restrictive versus liberal blood transfusion for gastrointestinal bleeding: a systematic review and meta-analysis of randomised controlled trials. Lancet Gastroenterol Hepatol.

[12] Rockey DC, Hafemeister AC, Reisch JS (2017). Acute on chronic gastrointestinal bleeding: A unique clinical entity. J Investig Med.

[13] Gelaw Y, Getaneh Z, Melku M (2021). Anemia as a risk factor for tuberculosis: a systematic review and meta-analysis. Environ Health Prev Med.

[14] Daves M, Cemin R, Zagler EM, Joos A, Platzgummer S, Hueber R (2016). Evaluation of capillary haemoglobin determination for anaemia screening in blood donation settings. Blood Transfus.

[15] Gómez-Escolar Viejo L, Soler Sala G, Palazón Azorin JM, Laudemia R, Sánchez J, Pérez-Mateo RM (2009). Fiabilidad de la medición de la hemoglobina por HemoCue® en pacientes con hemorragia gastrointestinal. Gastroenterol Hepatol.

[16] Bell S, Sweeting M, Ramond A, et al. Comparison of four methods to measure haemoglobin concentrations in whole blood donors (COMPARE): A diagnostic accuracy study. Transfus Med. 2021;31(2):94-103. 10.1111/tme.12750. 10.1111/tme.12750 PMC8048787 33341984

[17] Garcés J, Ródenas F (2015). La gestión de casos como metodología para la conexión de los sistemas sanitario y social en España. Aten Primaria.

[18] Morales-Asencio JM (2014). Gestión de casos y cronicidad compleja: Conceptos, modelos, evidencias e incertidumbres. Enferm Clin.

[19] Perteguer-Huerta I (2014). Case management: moving forward. Enferm Clin.

[20] Moher D. CONSORT: An Evolving Tool to Help Improve the Quality of Reports of Randomized Controlled Trials. JAMA. 1998;279(18):1489–91. 10.1001/jama.279.18.1489. 10.1001/jama.279.18.1489 9600488

[21] MiCoBioMed. Veri-Q RED. 2015; [update 2021 july 30] available from: http://www.micobiomed.com/en/product/product_view.php?part1_idx=2&part_idx=2&idx=22.

[22] Curtis AC, Keeler C (2021). Sampling Design in Nursing Research. Am J Nurs.

[23] Skevington SM, Lotfy M, O’Connell KA (2004). The World Health Organization’s WHOQOL-BREF quality of life assessment: psychometric properties and results of the international field trial A report from the WHOQOL Groupq. Qual Life Res.

[24] Luengo BT (2018). Hemoglobinómetro para la detección precoz de las anemizaciones. Actual en Med Fam.

